# SLC7A11: a new regulator in diabetic wound healing

**DOI:** 10.1038/s41392-022-01171-z

**Published:** 2022-09-14

**Authors:** Shunli Rui, Yu Ma, Wuquan Deng

**Affiliations:** Department of Endocrinology and Metabolism, Chongqing University Central Hospital, Chongqing Emergency Medical Center, Chongqing, China

**Keywords:** Endocrinology, Inflammation

In a recent paper published in *Nature*, Maschalidi and colleagues described the inhibition of SLC7A11 function in dendritic cells (DCs) as a crucial regulator for acceleration of wound healing in diabetes. The membrane transporter SLC7A11’s identification as a molecular brake on efferocytosis is important to better understand the role of DCs in regulating tissue repair, especially for diabetic wound healing.^1^

Chronic cutaneous wounds induced by inflammatory conditions impact on quality of life and constitute a substantial burden for people with diabetes.^2^ Efferocytosis refers to a form of apoptotic cells cleared by phagocytes around the wound edge to resolve inflammation and accelerate wound repair.^3^ Immune cells are an important function to maintain skin homeostasis. Macrophages and neutrophils have proved a closely link with wound healing dynamics.^4^ Nevertheless, the role of DCs in skin tissue regeneration is still unknown. Maschalidi et al. unexpectedly discovered that several SLC7 gene family members were upregulated via transcriptomics of efferocytic DCs in mouse. Among them, Slc7a5 and Slc7a11 were significantly increased. Moreover, Slc7a11 expression was consistently inhibited by DCs, resulting in increased efferocytosis, whereas inhibition of Slc7a5 via knockdown or pharmacological approach indicated an increasing trend for efferocytosis but without statistically significant difference. In addition, Slc7a11 in skin-resident DCs was most highly expressed through public database analysis and comparing them via Triwise plots. Further analysis, SLC7A11 colocalized with the DC but not fibroblasts in wounded skin tissue. Both innate immune cells and skin lysate of wounding expressed Slc7a11, along with wounding genes, such as Tgfb1 and Tnf. Therefore, SLC7A11 was selected as a new candidate target for the regulation of efferocytosis-mediated wound healing (Fig. 1a).

**Fig. 1:**
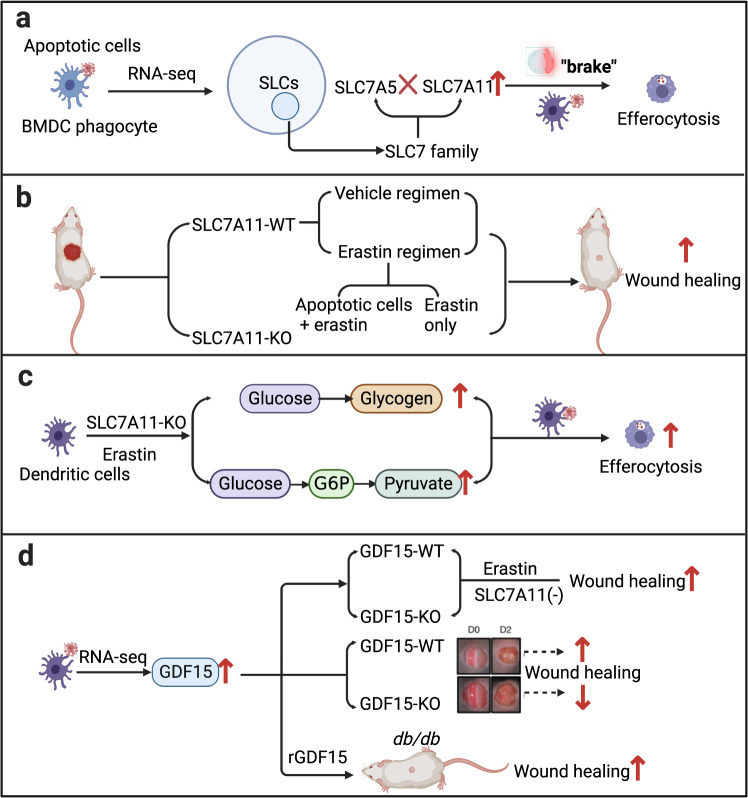
Boosting efferocytosis improves diabetic wound healing via targeting SLC7A11 in dendritic cells (DCs). **a** SLC7A11 acts as brake on DC efferocytosis, which was revealed by RNA-seq in DCs during efferocytosis. **b** Interfering with SLC7A11 function affected wound healing dynamics by erastin co-administering early-stage apoptotic cells in vivo. **c** Efferocytosis in DCs enhanced via glycogenolysis with inhibition or knockout of SLC7A11 in vitro. **d** SLC7A11 inhibition at the wound site can boost diabetic wound healing by improving apoptotic cell clearance and GDF15 secretion. Created with BioRender.com

The authors surprisingly discovered that there is no improvement in wound healing after only administering imidazole ketone erastin 40 (IKE), a metabolically stable, water-soluble inhibitor of SLC7A11. While co-administering erastin combined with early-stage apoptotic cells, the wound could be closed up 50% at day 4, approximately 2 days earlier than only erastin or apoptotic cells treatment. Correspondingly, co-administration with apoptotic cells at the beginning of wounding also accelerated wound healing in SLC7A11-knockout mice. Thus, by inhabitation of SLC7A11 function, cutaneous wounds can be accelerated for healing in the presence of apoptotic cells at an early stage (Fig. 1b).

This prompted the authors to further explore the synergistic relationship between the loss/inhibition of SLC7A11 function and apoptotic cells. Compared with the wild-type DCs, 191 differentially expressed genes were in efferocytic SLC7A11-KO DCs, which are mainly related to metabolism, protein synthesis, transcriptional regulation, and wound healing. Glycolysis of glucose by aerobic bacteria is critical for efferocytosis. Among phagocytes, DCs are unique in that they store glycogen intracellularly. To figure out this, Maschalidi et al. investigated whether DCs deficient in SLC7A11 produced more glucose from their glycogen reserves, thereby promoting aerobic glycolysis. The results revealed that efferocytosis in DCs indeed enhanced via glycogenolysis with inhibition or knockout of SLC7A11. Overall, the results confirm that DCs with SLC7A11-KO or erastin treatment increase glycolysis in order to meet the bioenergetic needs of efferocytosis (Fig. 1c).

Nowadays, it is a big challenge to boost diabetic wound healing.^5^ The diabetic wound was delayed healing in a leptin receptor-deficient *db/db* mice in the present study. The expression of Slc7a11 in the wounds on day 4 was 200-fold higher compared with normoglycaemic C57BL/6 mice. Meanwhile, within 4 days of wounding, C57BL/6 mice had ~400 apoptotic cells; after 13 days, they had ~50 apoptotic cells, whereas those of diabetic mice retained ~400 apoptotic cells after 13 days. As a result, both erastin alone and erastin regimen can accelerate the healing of diabetic wounds. The *db/db* mice had a lower number of DCs than wild-type mice, and the difference was greater at earlier time points in ex vivo, which could be reversed by SLC7A11 inhibition with erastin (Fig. 1d).

Interestingly, supernatants purified from efferocytic SLC7A11-KO DCs were found to accelerate the wound closure in mice. For the purpose of identifying possible growth factors related to wound healing, the transcriptome of efferocytic SLC7A11-deficient DCs was analyzed. The results revealed that growth differentiation factor 15 (GDF15) was significantly up-regulated. On day 2 after excisional skin biopsy, wounds in GDF15-deficient mice were larger than in control mice, and this was associated with increased Slc7a11 expression. As expected, GDF15-deficient DCs showed similar levels of Slc7a11 expression and efferocytosis to control DCs, supporting the hypothesis that GDF15 is produced downstream of efferocytosis. A partial enhancement in wound closure was observed with erastin treatment in GDF15-KO mice, suggesting SLC7A11 inhibition can have both GDF15-dependent and DF15-independent effects. Diabetes-prone mice suffer from reduced efferocytosis and low GDF15 levels, both associated with high SLC7A11 expression, showing a significantly slower healing process. Hence, by inhibiting SLC7A11 at wound sites, diabetic wound healing can be enhanced through at least two mechanisms, including improved cell clearance and the release of factors such as GDF15 by efferocytic cells (Fig. 1d).

In summary, DCs-mediated efferocytosis in skin plays a vital role in cutaneous tissue regeneration/wound healing. This research from Maschalidi and colleagues provides a new perspective: SLC7A11 was identified as a brake on DC phagocytosis and provided an approach to improve diabetic wound healing via inhibition of SLC7A11. However, the possible molecular mechanism of a SLC7A11 brake in DCs is an intriguing conundrum. Increased efferocytosis would lead to excessive antigen presentation, potentially overstimulating the immune system and increasing autoimmune reactions. Based on the work of Maschalidi et al. and others, we believe further mechanistic study on immune cell through the process of efferocytosis will lead to successful wound healing in diabetes.

## References

[1] Maschalidi S (2022). Targeting SLC7A11 improves efferocytosis by dendritic cells and wound healing in diabetes. Nature.

[2] Armstrong D, Boulton A, Bus S (2017). Diabetic foot ulcers and their recurrence. N. Engl. J. Med..

[3] Doran A, Yurdagul A, Tabas I (2020). Efferocytosis in health and disease. Nat. Rev. Immunol..

[4] Dardenne, C., et al. Topical aspirin administration improves cutaneous wound healing in diabetic mice through a phenotypic switch of wound macrophages toward an anti-inflammatory and pro-resolutive profile characterized by LXA4 release. *Diabetes*. 2022. 10.2337/db20-1245. 10.2337/db20-1245 35796692

[5] Rayman G (2020). Guidelines on use of interventions to enhance healing of chronic foot ulcers in diabetes (IWGDF 2019 update). Diabetes Metab. Res. Rev..

